# Humanizing π-Class Glutathione *S*-Transferase Regulation in a Mouse Model Alters Liver Toxicity in Response to Acetaminophen Overdose

**DOI:** 10.1371/journal.pone.0025707

**Published:** 2011-10-11

**Authors:** Matthew P. Vaughn, Debika Biswal Shinohara, Nicole Castagna, Jessica L. Hicks, George Netto, Angelo M. De Marzo, Traci J. Speed, Zachery R. Reichert, Bernard Kwabi-Addo, Colin J. Henderson, C. Roland Wolf, Srinivasan Yegnasubramanian, William G. Nelson

**Affiliations:** 1 Sidney Kimmel Comprehensive Cancer Center, Johns Hopkins University, Baltimore, Maryland, United States of America; 2 Department of Environmental Health Sciences, Bloomberg School of Public Health, Johns Hopkins University, Baltimore, Maryland, United States of America; 3 Howard University Cancer Center, Howard University, Washington, District of Columbia, United States of America; 4 Cancer Research United Kingdom Molecular Pharmacology Unit, Biomedical Research Institute, Ninewells Hospital and Medical School, Dundee, United Kingdom; Albert Einstein Institute for Research and Education, Brazil

## Abstract

**Background:**

Glutathione *S*-transferases (GSTs) metabolize drugs and xenobiotics. Yet despite high protein sequence homology, expression of π-class GSTs, the most abundant of the enzymes, varies significantly between species. In mouse liver, hepatocytes exhibit high *mGstp* expression, while in human liver, hepatocytes contain little or no *hGSTP1* mRNA or hGSTP1 protein. π-class GSTs are known to be critical determinants of liver responses to drugs and toxins: when treated with high doses of acetaminophen, *mGstp1/2+/+* mice suffer marked liver damage, while *mGstp1/2−/−* mice escape liver injury.

**Methodology/Principal Findings:**

To more faithfully model the contribution of π-class GSTs to human liver toxicology, we introduced *hGSTP1*, with its exons, introns, and flanking sequences, into the germline of mice carrying disrupted *mGstp* genes. In the resultant *hGSTP1+mGstp1/2−/−* strain, π-class GSTs were regulated differently than in wild-type mice. In the liver, enzyme expression was restricted to bile duct cells, Kupffer cells, macrophages, and endothelial cells, reminiscent of human liver, while in the prostate, enzyme production was limited to basal epithelial cells, reminiscent of human prostate. The human patterns of *hGSTP1* transgene regulation were accompanied by human patterns of DNA methylation, with bisulfite genomic sequencing revealing establishment of an unmethylated CpG island sequence encompassing the gene promoter. Unlike wild-type or *mGstp1/2−/−* mice, when *hGSTP1+mGstp1/2−/−* mice were overdosed with acetaminophen, liver tissues showed limited centrilobular necrosis, suggesting that π-class GSTs may be critical determinants of toxin-induced hepatocyte injury even when not expressed by hepatocytes.

**Conclusions:**

By recapitulating human π-class GST expression, *hGSTP1+mGstp1/2−/−* mice may better model human drug and xenobiotic toxicology.

## Introduction

Cytosolic glutathione *S*-transferases (GSTs) are encoded by a superfamily of genes grouped intoα, μ, π, σ, θ, ζ, and ω classes by primary amino acid sequence [1], [2]. The enzymes, which can exist as homo- or heterodimers of subunit polypeptides, catalyze reactions involving the conjugation of reduced glutathione (GSH) to electrophilic substrates. By targeting electrophiles generated by cytochrome P450s for conjugation with GSH, GSTs contribute to coordinated drug and xenobiotic metabolism, promoting the ultimate elimination of potential toxins by the ATP-dependent glutathione *S*-conjugate export pump [3]. The π-class GST, a homodimeric enzyme which was first described as a placental isoform, has since been found in many different tissues and is now known to be the most abundant of the GSTs [4]. The amino acid sequence of π-class GST subunit polypeptides is remarkably conserved across species, perhaps because the enzymes have been implicated in a broad array of vital cell and tissue functions, including xenobiotic metabolism, cell signaling, mutagen/carcinogen defense, and antineoplastic drug resistance [5].

Human π-class GSTs are encoded by a single gene, *hGSTP1*, while mice have two such genes, *mGstp1* and *mGstp2*. Each of the genes has 7 exons that encode enzyme subunit polypeptides of 210 amino acids [5]. The degree of protein homology is striking: of the 210 amino acids, 170 (81%) are completely conserved, and accounting for conserved and semi-conserved substitutions, the amino acid sequence across species has a 94% homology. *hGSTP1*, *mGstp1*, and *mGstp2* also share some common transcriptional promoter elements, including an AP1 (TGA[C/G]TCA) transcription factor binding site and GC boxes (GGGCGG) permitting SP1 transcription factor binding just upstream of a TATA box. *hGSTP1* and *mGstp2* have two SP1 binding sites; *mGstp1* has only one. For *hGSTP1*, the AP1 site and at least one of the SP1 sites must be intact for *hGSTP1* transcription to occur [6], [7].

Despite the extensive coding sequence homology, the transcription promoter regions and introns display little similarity across species, aside from the AP1 and SP1 sites. *hGSTP1* contains several unique promoter elements that could contribute to transcriptional regulation, including a pentad B sequence repeated 18 to 21 times and an overlapping NF-κB and C/EBP site (TTAAGGGAATTTCC). Also noteworthy is the abundance of CpG dinucleotides (n = 39) found between an [ATAAA]_n_ repeat region and the *hGSTP1* transcription start site. These CpGs, which are unmethylated in normal cells, are targeted for *de novo* methylation in many human cancers, leading to somatic epigenetic silencing of transcription [8], [9].

As a consequence of the likely differences in transcriptional regulation, π-class GST expression patterns vary widely across species, and these expression differences appear to have functional consequences. As an example, shortly after the discovery of rGST-P, the rat π-class GST, levels of the enzyme were found to be stereotypically elevated in preneoplastic hepatic foci, and in neoplastic lesions, induced by the chemical carcinogens diethylnitrosamine (DEN) and 2-acetylaminofluorene (AAF) [10]. Because rGST-P is not expressed in rat hepatocytes under normal conditions, the appearance of rGST-P-expressing cells has emerged as a reliable biomarker of hepatocarcinogenesis in the rat [11], [12]. However, this rat model has been difficult to extend to mice, which exhibit constitutive production of mGstp1/2 in normal hepatocytes, or to humans, which like rats fail to express π-class GSTs in hepatocytes, but do not induce π-class GST expression in preneoplastic or neoplastic lesions [13], [14], [15], [16], [17], [18], [19]. Of interest, many strains of mice appear much less susceptible than rats to liver carcinogenesis induced by chemical carcinogens [5], [20].

In an attempt to recapitulate human patterns of π-class GST expression in mice, we introduced the full-length *hGSTP1* gene, along with its *cis*-transcriptional regulatory sequences, into the germline of mice carrying disrupted *mGstp* genes. The resultant “humanized” strain of *hGSTP1+mGstp1/2−/−* mice showed marked differences from wild-type *mGstp1/2+/+* mice in the expression of π-class GSTs in liver tissues, with an absence of the enzymes in hepatocytes that was reminiscent of the lack of enzymes in human hepatocytes, but maintenance of expression in bile duct cells, Kupffer cells, macrophages, and endothelial cells. These expression differences were responsible for functional differences in liver toxicology, with *hGSTP1+mGstp1/2−/−* mice exhibiting far less liver injury than *mGstp1/2+/+* mice upon administration of high doses of acetaminophen [21]. Nonetheless, *hGSTP1+mGstp1/2−/−* mice did manifest limited centrilobular necrosis not seen in *mGstp1/2−/−* mice, suggesting that π-class GSTs remained critical determinants of liver damage even when not present in hepatocytes.

## Results

### π-class GST expression in hGSTP1+ mGstp1/2−/− mice

**Figure 1:**
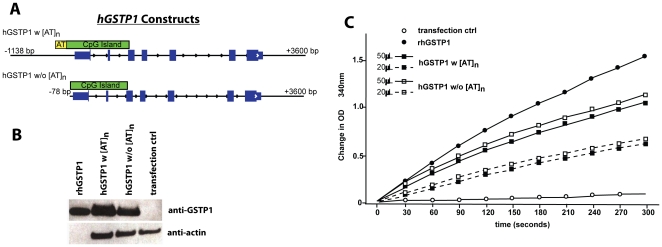
Full length *hGSTP1* directs expression of catalytically-active GSTπ. When transfected into Hep3B cells, which do not express *hGSTP1* mRNA or hGSTP1 polypeptides, (A) both full length *hGSTP1* (−1138 to +3600) and *hGSTP1* with [ATAAA]_n_ ([AT]_n_) repeat sequences deleted (−78 to +3600) directed expression of (B) GSTP1 polypeptides, as detected by immunoblot analysis, and (C) active GSTπ, as detected by CDNB assay using the indicated volumes of HEP3B cell lysates, with appearance of the glutathione conjugate of CDNB monitored as change in OD_340 nm_. (A–C) Yellow box, position of [AT]n repeat; Green box, position of CpG island; positions are shown with respect to the hGSTP1 transcriptional start site; blue boxes, position of exons; rhGSTP1, recombinant human GSTP1 polypeptides *in vitro*. Transfection controls directed expression of GFP polypeptides.

To create humanized *hGSTP1+ mGstp1/2−/−* mice, a linearized DNA fragment containing full length *hGSTP1* gene (−1138 to +3600) was microinjected into C57BL/6 mouse oocytes, yielding some 16 offspring. Before attempting to target this gene to the germline of mice, this construct or a 5′ deletion construct with truncated 5′ regulatory regions lacking the [ATAAA]_n_ pentad repeat (Fig. 1A), was transfected into Hep3B human liver cancer cells, which are normally devoid of π-class GSTs as a result of epigenetic silencing of *hGSTP1* attributable to somatic CpG island hypermethylation [16]. *hGSTP1* transfection promoted expression of π-class GST subunit polypeptides and an increase in GST activity (Fig. 1B–C). Three *hGSTP1+* founder mice were then crossed to C57BL/6 *mGstp1/2−/−* mice; after two generations of breeding the resultant *hGSTP1+mGstp1/2−/−* mouse strain was maintained by breeding with *mGstp1/2−/−* mice and selecting progeny carrying *hGSTP1+* alleles. *hGSTP1+mGstp1/2−/−* mice were not grossly different in appearance or behavior from *mGstp1/2+/+* or *mGstp1/2−/−* mice.

### Patterns of π-class GST expression differ substantially between humans and mice

**Figure 2:**
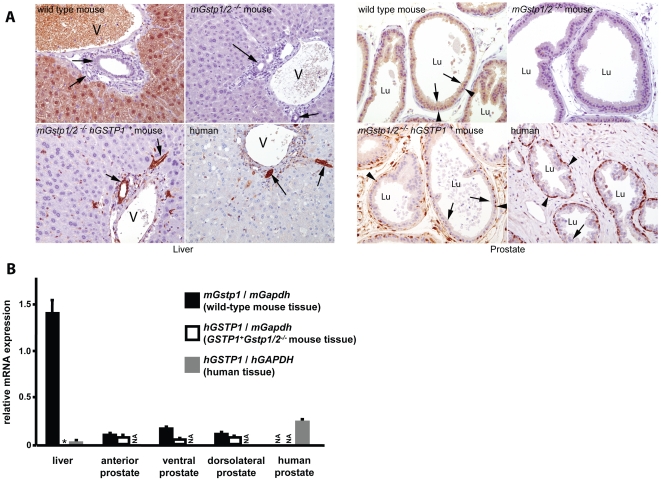
Regulation of π-class GST expression in *hGSTP1+mGstp1/2−/−* mice. (A) Immunohistochemistry using anti-GSTπ antibodies for liver and prostate tissues from a wild type mouse, *mGstp1/2−/−* mouse, *hGSTP1+mGstp1/2−/−* mouse, and human. Left panels show liver staining; arrows, bile ducts; V, central vein. Right panels show prostate staining, arrows, representative luminal cells; arrowheads, representative basal cells; Lu, lumen of prostate gland acini. All images are 200× original magnification. (B) Quantitative RT-PCR for *mGstp1* and *hGSTP1*, normalized to *mGapdh* or *hGAPDH*, using RNA from various tissues.

To ascertain whether human π-class GST expression patterns were recapitulated in mice by transfer of *hGSTP1*, with all of its known *cis*-regulatory sequences, various tissues from mice and from humans were subjected to immunohistochemical staining analyses of enzyme content using anti-π-class GST antibodies (Fig. 2A). For liver tissues, in *mGstp1/2+/+*mice, π-class GSTs were present in the nucleus and cytoplasm of hepatocytes, but not prominently in Kupffer cells, bile duct cells, and endothelial cells, while in *hGSTP1+ mGstp1/2−/−* mice, enzyme expression was restricted to non-hepatocytes, including endothelial cells, Kupffer cells, and bile duct cells. In this way, the pattern of π-class GST expression in human liver, where the hepatocytes are devoid of the enzymes but show expression in bile duct, endothelial, and Kupffer cells, was better recapitulated in *hGSTP1+mGstp1/2−/−* mice than in *mGstp1/2+/+*mice. Human prostate tissues produce π-class GSTs predominantly in basal epithelial cells, and much less so in luminal epithelial cells. As in the liver, this human pattern of expression was better recapitulated in *hGSTP1+mGstp1/2−/−* mice than in *mGstp1/2+/+*mice. Furthermore, the antibodies used could detect both mouse and human π-class GST with great specificity, as no immunoreactive peptides were evident in tissues from *mGstp1/2−/−* mice.

Next, mRNAs from various tissues from mice and from humans were subjected to quantitative RT-PCR for *mGstp1*, *hGSTP1*, *mGapdh*, and *hGAPDH* (Fig. 2B). In tissues from mature male C57BL/6 wild type mice, *mGstp1* mRNA was present at high levels in the liver, relative to *hGSTP1* mRNA, and expressed at lower levels in the kidney and in each of the lobes of the prostate. In contrast, in human tissues, *hGSTP1* mRNA, relative to *hGAPDH*, was barely detectable in the liver, but expressed at low levels in the kidney and prostate. For *hGSTP1+mGstp1/2−/−* mice, *hGSTP1* mRNA expression patterns, relative to *mGSTP1* mRNA, resembled that of *hGSTP1* mRNA, relative to *hGAPDH*, in human tissues: *hGSTP1* mRNA was nearly absent from the liver while present in the kidney and in each of the prostate lobes. Of note, anatomically distinct mouse prostate lobes may be analogous to non-anatomically distinct human prostate zones, so each of the mouse lobes were considered independently, while the human prostate was assessed as a single entity.

### GSTP1 CpG island DNA methylation patterns in hGSTP1+ mice

**Figure 3:**
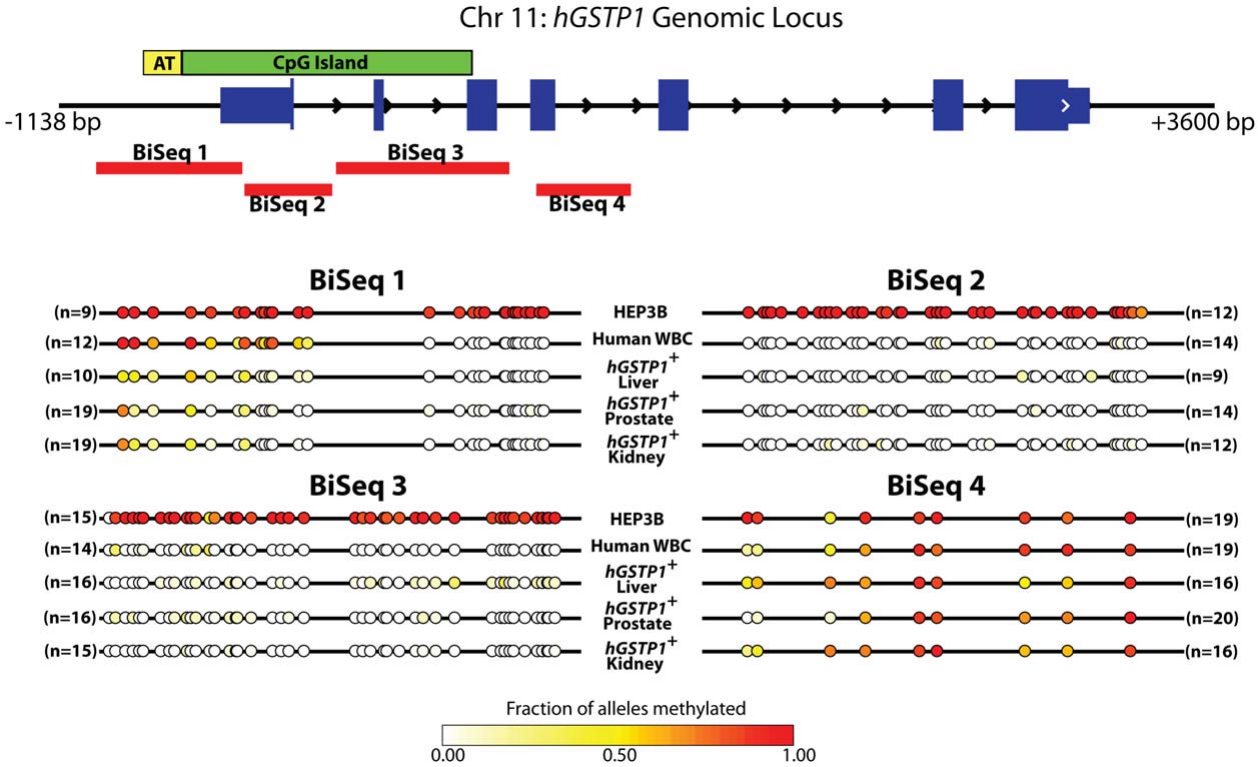
The hGSTP1 transgene in hGSTP1+mGstp1/2−/− mice recapitulates the pattern of methylation seen in normal human cells. Bisulfite genomic sequencing for ^5-me^C in four amplicons (labeled BiSeq 1–4)from the *hGSTP1* CpG island using DNA from various *hGSTP1+mGstp1/2−/−* mouse tissues (*hGSTP1*+ liver, prostate, kidney), along with DNA from human white blood cells (normal *hGSTP1* ^5-me^C pattern) and Hep3B cells (hypermethylated at *hGSTP1* CpG island). Each row represents a summary of multiple independently cloned alleles from each sample as indicated. Circles indicate the relative position of CpG dinucleotides within the bisulfite sequencing amplicon. The color of each circle is scaled to the fraction of alleles methylated for the indicated CpG according to the heatmap shown at the bottom.

In cancers arising in human liver and human prostate, absence of π-class GST expression is a consequence of somatic CpG methylation changes at a CpG island sequence encompassing the *hGSTP1* transcriptional regulatory region [8], [16]. Remarkably, though the gene microinjected into mouse oocytes was without any ^5-me^C bases, when maintained in the germline, the *hGSTP1* alleles present in *hGSTP1+ mGstp1/2−/−* mice established patterns of CpG dinucleotide methylation in adult cells that was similar to those seen in *hGSTP1* in normal adult human cells (Fig. 3) [22]. This suggested that *cis*-elements in *hGSTP1* were not only sufficient to establish human patterns of hGSTP1 expression in *hGSTP1+ mGstp1/2−/−* mice, but also sufficient to establish human patterns of CpG dinucleotide/CpG island methylation. Finally, the lack of expression of π-class GSTs in hepatocytes and prostate luminal cells in *hGSTP1+ mGstp1/2−/−* mice could not be explained by hypermethylation of *hGSTP1* CpG island sequences.

### Toxicity of acetaminophen (APAP)

**Figure 4:**
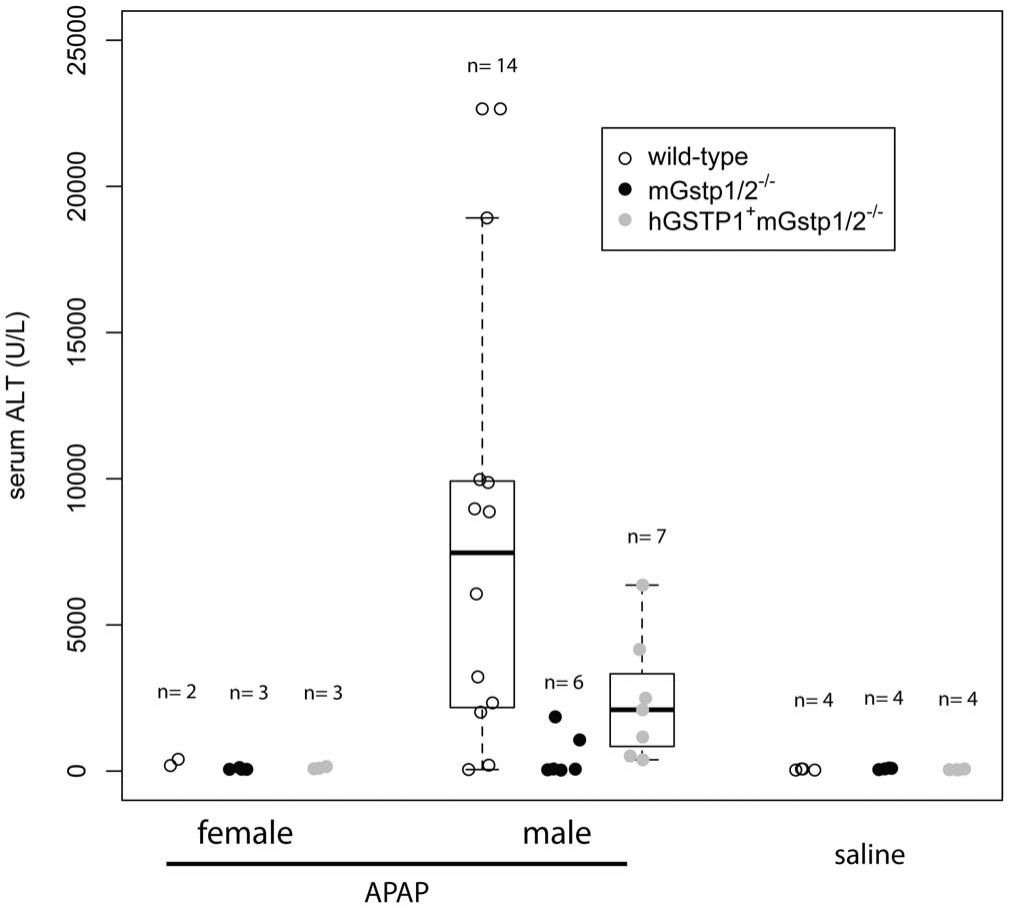
APAP-induced liver injury in male and female wild type, *mGstp1/2−/−*, and *hGSTP1+mGstp1/2−/−* mice. Serum ALT levels 24 hours after intraperitoneal injection of 300 mg/kg APAP or saline are shown. Compared to mGstp1/2−/− mice, mGstp1/2+/+ (p<0.04) and hGSTP1+mGstp1/2−/− (p<0.03) mice exhibited significantly higher ALT elevations in response to APAP overdose. Data was analyzed by Wilcoxon Rank Sum test.

**Figure 5:**
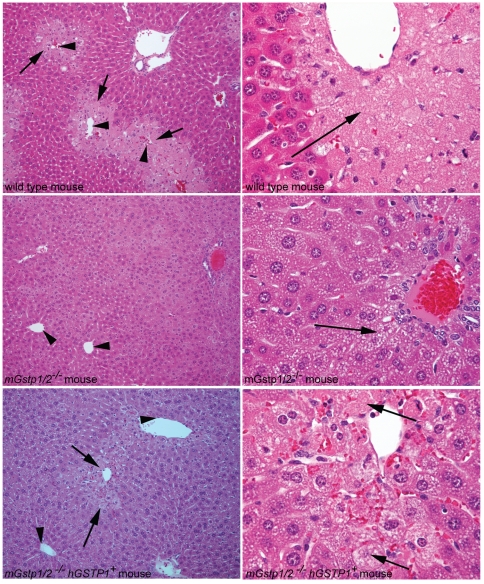
Histopathology of liver tissues from APAP-treated wild type, *mGstp1/2−/−*, and *hGSTP1+mGstp1/2−/−* mice. Arrowheads denote central veins; arrows highlight liver injury/necrosis. Left panels are 200× original magnification and Right panels are 400× original magnification.

Data obtained from *in vitro* studies using purified enzymes suggested that π-class GSTs might be major contributors to the liver disposition of acetaminophen (*N*-acetyl-*p*-aminophenol; APAP) by catalyzing reactions between its reactive metabolite *N*-acetyl-*p*-benzoquinoneimine (NAPQI) and glutathione [23]. However, when *mGstp1/2−/−* mice were challenged with an overdose of acetaminophen (APAP), the mice exhibited a marked decreased, not increased, hepatotoxicity when compared to *mGstp1/2+/+* mice [21]. To ascertain whether in *hGSTP1+mGstp1/2−/−* mice, which like *mGstp1/2−/−* mice, fail to produce π-class GSTs in hepatocytes, were sensitive or resistant to liver damage caused by APAP, we treated *mGstp1/2+/+* mice, *hGSTP1+mGstp1/2−/−* mice, and *mGstp1/2−/−* mice with 300 mg/kg APAP and assessed the mice for liver damage using serum assays for alanine aminotransferase (ALT) and histopathology examination of liver tissues (Fig. 4, 5). Male *mGstp1/2+/+* mice that had been fasted for 24 hours and then given APAP by intraperitoneal injection exhibited marked increases in serum ALT levels relative to saline-treated controls (p<0.04) and also showed massive centrilobular and bridging necrosis in the liver with around 40–50% hepatocyte death. In contrast, *mGstp1/2−/−* mice showed very little evidence of liver damage in response to APAP, and no significant differences in serum ALT levels. The humanized *hGSTP1+mGstp1/2−/−* mice treated with APAP showed low levels of liver injury, with small serum ALT elevations, more limited centrilobular necrosis without bridging, and mild hepatocellular necrosis (less than 10%). ALT levels were significantly greater than those observed in the APAP-treated *mGstp1/2−/−* mice (p = 0.03), but lower than wildtype mice. As previously reported, significant gender differences were observed in response to the acetaminophen treatment in mGstp1/2+/+ mice. Female wildtype mice did not show significant elevations in ALT following acetaminophen treatment relative to saline-treated control. This gender bias in APAP mediated ALT elevations was also present in the acetaminophen-treated hGSTP1+mGstp1/2−/− mice, which also did not show significant elevations in ALT relative to saline-treated controls. In fact, regardless of genotype, female mice did not exhibit elevations in ALT following acetaminophen treatment.

**Figure 6:**
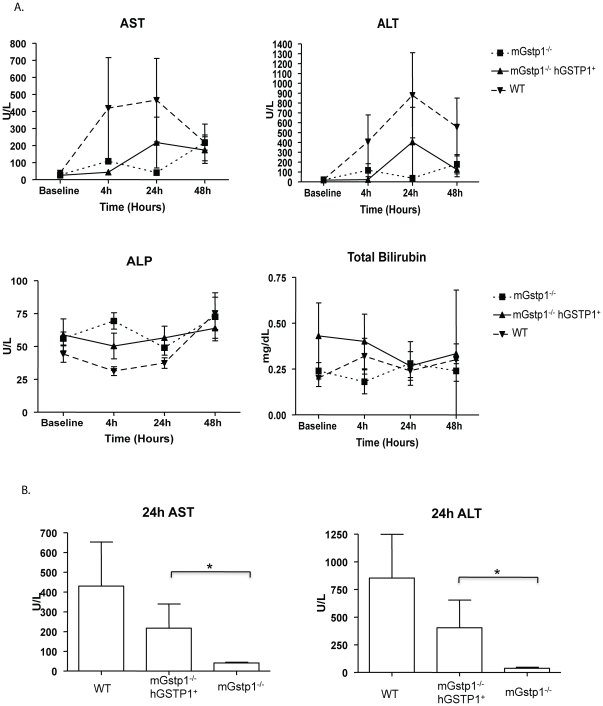
Acetaminophen-induced hepatic injury in male wildtype, mGstp1/2−/−, and hGSTP+mGstp1/2−/− mice. Baseline serum measurements were taken prior to administration of the drug. Mice were fasted for 12 or 24 hours and then administered vehicle or 300 mg/kg acetaminophen via gavage. (A) Serum AST, ALT, ALP and total bilirubin measurements taken in serum collected at 4, 24, and 48 hours. (B) Serum measurements of AST and ALT at 24 hours after acetaminophen administration. N = 5 mice per group * = p<0.05 by Students t-test.

In a second experiment, mice were administered 300 mg/kg acetaminophen or vehicle by oral gavage in order to more faithfully model human exposure to the drug. Serum markers of parenchymal liver damage (as measured by ALT and AST) as well as biliary duct damage (as measured by alkaline phosphatase and total bilirubin) were assessed at various timepoints (Fig. 6A). In wildtype mice levels of ALT and AST increased relative to baseline measurements 4 hours after drug administration and peaked at 24 hours. In contrast, *mGstp1/2^−/−^* mice were impervious to acetaminophen administration, and serum markers remained normal. Interestingly, *hGSTP1^+^mGstp1/2^−/−^* mice exhibited an intermediate level of sensitivity to acetaminophen relative to the wildtype and *mGstp1/2^−/−^* mice – elevations of AST and ALT at 24 hours in hGSTP1+mGstp1/2−/− mice were significantly greater than those in mGstp1/2−/− mice but showed a trend to be lower than those seen in wildtype mice (Figure 6A–B). Acetaminophen treatment did not result in increases in serum levels of total bilirubin or alkaline phosphate outside of the normal range, suggesting that acetaminophen-induced damage was primarily occurring in the hepatocytes.

## Discussion

The generation of a “humanized” hGSTP1-transgenic mouse strain has created a new mouse model for the study of human metabolism, toxicology, and carcinogenesis. The different patterns of π-class GST expression in *mGstp1/2+/+* mice and *hGSTP1+mGstp1/2−/−* mice, with accompanying differences in response to drugs like acetaminophen, may provide new insights not just into the biochemistry of drug and toxin disposition, but rather the cell and tissue specific responses to stress and injury inflicted by such agents. In this way, *hGSTP1+mGstp1/2−/−* mice can join an increasing family of “humanized” transgenic mice created to better understand the xenobiotic metabolism [24].

To make such “humanized” transgenic mice, three general approaches have been pursued. One technique involves the construction of transgenes containing cDNA encoding human metabolizing enzymes ligated to transcription regulatory sequences to drive cell- or organ-specific enzyme expression. A second strategy features recombination (or “knock-in”) of human coding sequences into an orthologous mouse gene. For mice generated in this way, human enzymes can be produced in a mouse pattern of expression. To create mice in which human enzymes are present in a human pattern of expression, introduction of the entire human gene into the germline of mice, as was done for *hGSTP1*, may be required. This last approach relies upon the recognition of human transcriptional regulatory sequences by murine transcription factors, which can direct chromatin assembly and lineage-specific gene expression. For *hGSTP1+mGstp1/2−/−* mice, the observed patterns of *hGSTP1* expression were most consistent with significant conservation in transcriptional *trans*-regulation between human and mouse. Finally, for any of the “humanized” transgenic mice producing human enzymes, there is a concern that simultaneous expression of both human and mouse enzymes might interfere generally with metabolic processes. The availability of gene knockout strains, such as *mGstp1/2−/−* mice, for breeding crosses with mice carrying human transgenes can reduce this worry.

One observation made during the expansion of the *hGSTP1+mGstp1/2−/−* mouse colony was a general increase in litter size of the *hGSTP1+mGstp1/2−/−* mice as compared to *mGstp1/2−/−* mice. The mechanistic basis for this difference has not been established. However, π-class GSTs were originally assigned the “P” (or “π”) family designation because the enzymes were first found in the placenta [4]. Perhaps, π-class GSTs provide some sort of fetal development or survival advantage, whether mouse or human. This advantage may also be manifest in a greater than ∼60% transmission of the *hGSTP1* transgene on the *mGstp1/2−/−* background (the expected frequency is 50%). Aside from the slight increase in litter size and gene transmission, there were no other obvious gross phenotypes in the *hGSTP1+mGstp1/2−/−* mouse strain.

When subjected to APAP overdoses, *hGSTP1+mGstp1/2−/−* mice responded differently than *mGstp1/2+/+* mice. There were also notable differences between the serum ALT levels of male and female mice after administration of acetaminophen. This phenomenon is in agreement with previous reports [25], [26] and is likely due to gender-dependent differences in expression of π-class GSTs; female mice have significantly lower expression of GST-π and are resistant to damage [21]. Despite intensive study, the mechanism(s) by which APAP, and APAP metabolism, cause liver injury has not been fully elucidated. Although APAP can be converted by cytochrome P450 enzymes to NAPQI, a reactive intermediate capable of inflicting significant damage, NAPQI can be detoxified by conjugation to GSH [27], [28]. The notion that π-class GSTs might afford protection against NAPQI generation from an overdose of APAP was undermined by the finding of a decrease, rather than increase, in liver injury accompanying APAP poisoning in *mGstp1/2−/−* versus *mGstp1/+/+* mice, a phenomenon confirmed by the current study [21]. Remarkably, between *mGstp1/2−/−* and *mGstp1/+/+* mice treated with APAP there were no differences in GSH depletion, though GSH levels recovered more quickly in *mGstp1/2−/−* mice. In addition, the difference in toxicity could not be attributed to differential expression of enzymes, such as CYP2E1 and other Phase I enzymes, responsible for the metabolism to acetaminophen to NAPQI [21], [29]. This π-class GST effect on APAP hepatotoxicity was reflected in the pattern and amount of damage generated by APAP overdose in *hGSTP1+mGstp1/2−/−* mice, which express little, if any π-class GSTs in hepatocytes, but exhibit abundant expression in bile duct cells, Kupffer cells, macrophages, and endothelial cells. Taken together, the data strongly suggest that π-class GSTs act to promote liver injury both by intrinsically augmenting hepatocyte death when expressed in hepatocytes, and also by indirectly triggering hepatocyte death when present in other liver cell types. One possibility for the intrinsic mechanism may be the influence of π-class GSTs on intracellular signaling pathways, such as the c-Jun N-terminal kinase (Jnk) pathway typically activated by cellular stress and proinflammatory cytokines [30]. In wild-type mice, APAP has been found to trigger prolonged activation of the Jnk pathway that contributes to hepatocyte death [31]. mGstp1/2−/− mice have been shown to have significantly higher basal levels of Jnk signaling compared wild type mice, but show only modest, if any, increase from this basal signaling after exposure to acetaminophen [29]. π-class GSTs, encoded by genes responsive to AP-1 transcription factors (and thus Jnk signaling), therefore interfere with constitutive Jnk activation, providing an autoregulatory loop that may be perturbed differently in the hepatocytes from *mGstp1/2+/+*, *mGstp1/2−/−*, and *hGSTP1+mGstp1/2−/−* mice subjected to the oxidative stress of APAP overdose, and this may partly underlie the differential levels of acetaminophen toxicity seen in these mice. The mechanism by which π-class GSTs present in bile duct cells, Kupffer cells, macrophages, and endothelial cells can trigger limited centrilobular necrosis in hepatocytes devoid of the enzyme is less clear. Nonetheless, centrilobular necrosis constitutes a common mode of human liver injury in response to liver toxin exposure. Perhaps, *hGSTP1+mGstp1/2−/−* mice may better model this type of liver response to toxins than wild-type mouse strains which contain high levels of π-class GSTs in hepatocytes.

Finally, the availability of *hGSTP1+mGstp1/2−/−* mice may permit new studies of carcinogenesis that better recapitulate human cancer development. By comparing responses of *mGstp1/2+/+* and *Gstp1/2−/−* mice to chemical carcinogens, π-class GSTs have been implicated in protection against tumor development in several different organ sites, including the skin, the colon, and the lungs [32], [33], [34]. For human cancers, the contributions of π-class GSTs appear to be more complex. During the pathogenesis of some human cancers, such as cancers of the colon, lung, kidney, and stomach, hGSTP1 has been reported to be expressed at high levels and contribute to anti-neoplastic drug resistance [14], [35]. In other human cancers, such as cancers of the prostate, liver, and breast, hGSTP1 is conspicuously absent [8], [16], [36]. Absence of hGSTP1 expression appears mostly attributable to somatic epigenetic *hGSTP1* silencing attributable to *de novo* changes in DNA methylation at a CpG island encompassing the gene promoter. Epigenetic silencing of π-class GST expression has not been reported for either *mGstp1/2* or for *rGST-P*. Since *hGSTP1* contains a much higher density of CpG dinucleotides than either of the rodent orthologs, perhaps *hGSTP1* may be more vulnerable than *mGstp1/2* or for *rGST-P* to epigenetic silencing during carcinogenesis. This hypothesis can be tested using *hGSTP1+mGstp1/2−/−* mice, which have established both a more human pattern of hGSTP1 expression in the liver and prostate and a human pattern of ^5-me^CpG distribution at the *hGSTP1* CpG island.

## Materials and Methods

### Ethics Statement

#### Animal Studies

All experimental protocols were approved and performed in accordance with the standards established by the U.S. Animal Welfare Acts, as set forth in the National Institutes of Health guidelines and in the Policy and Procedures Manual of the Johns Hopkins University Animal Care and Use Committee (Approval Numbers MO10M447, MO08M163, MO08M465, MO08M487).

#### Human Samples

Human tissues and nucleic acids were obtained and used following protocols and guidelines approved by the Johns Hopkins University Institutional Review Board (Study number: NA_00048544). For bisulfite sequencing analysis, human male genomic DNA was obtained from Novagen (Cat # 70572).

### Generation of hGSTP1 transgenic mice

The full length *hGSTP1* gene (−1138 to +3600) was amplified from a bacterial artificial chromosome (BAC) containing sequence from chromosome 11q (accession number AP001184; from BACPAC Resource Center (BPRC) at Children's Hospital Oakland Research Institute , Oakland, CA) using *Pfu*Ultra polymerase (Stratagene). A plasmid containing full length *hGSTP1*, along with a plasmid that had the 5′ [ATAAA]_n_ repeat sequences deleted (−78 to +3600), were transfected into Hep3B cells, known to be devoid of *hGSTP1* mRNA and hGSTP1 polypeptides as a result of epigenetic *hGSTP1* silencing, to ensure that the gene was capable of directing expression of catalytically-active GSTπ [16]. Transfection was accomplished using Lipofectamine Plus (Invitrogen) in serum free minimal essential medium (MEM). The full length *hGSTP1*-containing construct was then provided to Xenogen (Hopkinton, MA) for pronuclear microinjection and development of the founder mice on a C57BL/6 strain background. To identify *hGSTP1+* progeny, genotyping specifically for *hGSTP1*, even in the presence of wild type mouse DNA, was accomplished by PCR using the forward primer 5′-AGGCGTGCAGATCACCTAAG-3′ and the reverse primer 5′- GCCACATCTGGCTGATTTTT-3′ with cycling conditions of 95°C for 3 minutes, 35 cycles of 95°C for 30 seconds, 59°C for 30 seconds, and 72°C for 45 seconds, yielding a 100 base pair product.

### Breeding to create hGSTP1+Gstp1/2−/− mice

All mice were housed in a pathogen-free environment, allowed free access to food and water, and were maintained on a 12 hour light/dark cycle. *mGstp1/2*−/− that had been extensively backcrossed to C57BL/6 mice were available for breeding crosses with *hGSTP1+* mice. Genotyping for *mGstp1/2+/+* and *mGstp1/2*−/− was performed using the same PCR reaction conditions as for *hGSTP1*, but with the following primers: *mGstp1/2+/+* forward 5′-GGCCACCCAACTACTGTGAT -3′, *mGstp1/2+/+* reverse 5′-AGAAGGCCAGGTCCTAAAGC -3′, *mGstp1/2*−/− forward 5′-CTGTAGCGGCTGATGTTGAA -3′, and *mGstp1/2*−/−reverse 5′-ATGGCGATTACCGTTGATGT -3′. To assess patterns of π-class GST expression and DNA methylation, mice were euthanized in a CO_2_ chamber according to Johns Hopkins Animal Care and Use Committee (ACUC) guidelines. After sacrifice, tissues were harvested and immediately placed in formalin or snap frozen in liquid nitrogen for further processing.

### Assessment of π-class GST expression by quantitative RT-PCR, immunoblot analysis, immunohistochemistry, and enzyme activity assay

RNA isolated from various human and mouse tissues was assayed for the expression of specific mRNAs using a QuantiTect Probe RT-PCR Kit (Qiagen, Valencia, CA) and primer sets for *hGSTP1*, *hGAPDH*, *mGstp1*, *mGstp2*, and *mGAPDH* from Applied Biosystems (Foster City, CA). π-class GST subunit polypeptide expression was detected in protein lysates from cells and tissues by immunoblot analysis with anti-GSTπ antibodies as previously described [8]; GST activity in the lysates was monitored using 1-chloro-2,4-dinitrobenzene (CDNB) as a substrate, with recombinant human GSTπ (Calbiochem) as a positive control [37]. Human and mouse tissues were evaluated for patterns of π-class GST expression via immunohistochemical staining of formalin fixed, paraffin embedded, tissue sections. Briefly, the sections were deparaffinized by rinsing in xylene and then in a gradient of ethanols (100%→70%) until clear; antigen retrieval was accomplished by citrate steaming for 20 minutes. After incubating for 5 minutes in hydrogen peroxide and rinsed, the slides were probed with anti-GSTπ antibody (Stressgen, Ann Arbor, MI) at a 1∶1500 dilution, overnight at 4°C. Antibody binding was revealed using a horseradish peroxidase-conjugated secondary antibody (Dako, Denmark) applied to tissue sections for 30 minutes. For color generation and counterstaining, slides were first incubated in diaminobenzidine (Sigma), and then in hematoxylin (Dako).

### Bisulfite genomic sequencing for assessment of hGSTP1 CpG island methylation

Sequence mapping of ^5-me^C bases at the *hGSTP1* CpG island was undertaken using bisulfite conversion methods and PCR primers for four amplicons as described by Millar *et al.* [22].

***Toxicology analyses.*** Mice were administered acetaminophen (*N*-acetyl-*p*-aminophenol; APAP; Sigma) via intraperitoneal (IP) injection or via oral gavage. APAP was used at a concentration of 300 mg/kg in phosphate buffered saline (PBS) and injections were undertaken using mice that were fasted for 12 hours prior to injection. Acetaminophen dosage was selected based on previous reports of hepatotoxicity in mice [21], [38]. To assess the injurious effects of APAP on liver histology and function, serum was collected from mice via submandibular venipuncture or cardiac puncture (at terminal timepoints). Serum samples were taken at 4, 24, and 48 hours after gavage. Mice were sacrificed via cardiac puncture/exsanguination following administration of 14 mg Avertin (20 mg/ml, Sigma) or via CO_2_ asphyxiation. The blood was collected in BD Microtainer SST tubes (BD, Franklin Lakes, NJ), allowed to clot for at least 1 hour at room temperature, and then centrifuged at 16,000× *g* for 2 minutes at 4°C to allow separation of serum. Serum specimens were assayed for alanine aminotransferase (ALT), aspartate aminotransferase (AST), alkaline phosphatase (ALP) and total bilirubin levels using VetAce (Alfa Wassermann, West Caldwell, NJ). For histopathology, dissected liver tissues were fixed in buffered formalin, embedded in paraffin, and then processed for tissue section staining with hematoxylin and eosin.
